# Risk of COVID-19 infection and the associated hospitalization, ICU admission and mortality in opioid use disorder: a systematic review and meta-analysis

**DOI:** 10.1186/s13722-022-00349-8

**Published:** 2022-11-30

**Authors:** Amir Hossein Behnoush, Elham Bazmi, Mehdi Forouzesh, Behnam Behnoush

**Affiliations:** 1 School of Medicine, Tehran University of Medical Sciences, Tehran, Iran; 2 Legal Medicine Research Center, Legal Medicine Organization, Tehran, Iran; 3 Department of Forensic Medicine and Toxicology, Tehran University of Medical Sciences, Tehran, Iran

**Keywords:** COVID-19, Opioid, Hospitalization, ICU, Mortality

## Abstract

**Background:**

Opioid use disorder (OUD) as a common drug use disorder can affect public health issues, including the COVID-19 pandemic, in which patients with OUD may have higher risk of infection and severe disease. This systematic review and meta-analysis was conducted to investigate the risk of COVID-19 and the associated hospitalization, intensive care unit (ICU) admission, and mortality in patients with OUD.

**Materials and Methods:**

A comprehensive systematic search was performed on PubMed, Scopus, Embase, and Web of Science to find studies which compared the infection rate and outcomes of COVID-19 in OUD patients in comparison with the normal population. A random effects meta-analysis model was developed to estimate odd ratios (OR) and 95% confidence interval (CI) between the outcomes of COVID-19 and OUD.

**Results:**

Out of 2647 articles identified through the systematic search, eight were included in the systematic review and five in the meta-analysis. Among 73,345,758 participants with a mean age of 57.90 ± 13.4 years, 45.67% were male. The findings suggested no significant statistical relationship between COVID-19 infection and OUD (OR (95% CI): 1.18 (0.47–2.96), p-value: 0.73). Additionally, patients with OUD had higher rate of hospitalization (OR (95% CI) 5.98 (5.02–7.13), p-value<0.01), ICU admission (OR (95% CI): 3.47 (2.24–5.39), p-value<0.01), and mortality by COVID-19) OR (95% CI): 1.52(1.27–1.82), pvalue< 0.01).

**Conclusion:**

The present findings suggested that OUD is a major risk factor for mortality and the need for hospitalization and ICU admission in patients with COVID-19. It is recommended that policymakers and healthcare providers adopt targeted methods to prevent and manage clinical outcomes and decrease the burden of COVID-19, especially in specific populations such as OUD patients.

**Supplementary Information:**

The online version contains supplementary material available at 10.1186/s13722-022-00349-8.

## Background

In March 2020, the World Health Organization announced COVID-19 as a pandemic. A total of 462 million confirmed patients with COVID-19 and over 6 million deaths caused by the disease had been reported until March 2022 [1, 2]. Although respiratory depression seems to be the most serious complication of COVID-19 infection, other physical and mental disorders were observed in infected individuals, especially in cases with a high virus load, such as healthcare workers [3–6]. As a major threat to public health, the growing mortality and morbidity associated with COVID-19 require the evaluation of the risk factors for COVID-19-induced complications.

As a common drug use disorder, opioid use disorder (OUD) globally affects 40.5 million people and 510 cases per 100,000 [7]. Research suggests that OUD is a risk factor that exacerbates COVID-19 outcomes [8, 9]. The immunosuppressive effects exerted on respiratory and mental systems by the unhealthy use of opioids can increase the risk of infection with severe acute respiratory syndrome coronavirus 2 (SARS-CoV-2) and cause COVID-19-associated hospitalization, prolonged Intensive Care Unit (ICU) stay, adverse events and death. Moreover, decreased lung capacity caused by COVID-19 can worsen the condition associated with opioid overdose [10, 11]. The discrepancy is observed among the results associated with complications of COVID-19, such as hospitalization, ICU admission, and mortality in the studies [12–14]. However, despite the research performed on the effects of drug abuse on COVID-19 complications, the relationships between COVID-19 and OUD have rarely been addressed in the literature [15, 16]. Acquiring information about the risks of COVID-19 outcomes associated with OUD can therefore help determine patient risk and introduce evidence-based actions to clinicians and policymakers.

The present systematic review and meta-analysis aimed at examining the influence of OUD on the risk of infection with COVID-19 in the early phases of the pandemic. Hospitalization, ICU admission, and mortality were also investigated in OUD patients with COVID-19 compared with non-OUD ones.

## Methods

### Inclusion criteria and search strategy

The present systematic review and meta-analysis was conducted based on Preferred Reporting Items for Systematic Reviews and Meta-Analyses (PRISMA) guidelines [17]. Two authors, EB and BB, independently searched online databases of PubMed, Embase, Web of Science, and SCOPUS for articles published from January 2020 to December 2021. Inclusion criteria were the observational studies that investigated the rate of COVID-19 infection (either by Polymerase Chain Reaction (PCR) or clinical diagnosis by the physician) or outcomes (COVID-19-related hospitalization, ICU admission due to COVID-19, and mortality from it) of OUD patients compared with the overall population or the studies with substance use disorder (SUD) which included opioids in the total SUD definition. Exclusion criteria were non-English publications, case series recruiting below twenty patients, case reports, review articles, editorials, conference abstracts, nonclinical studies, preprints, and non-peer-reviewed studies, in addition to studies that did not mention OUD in their total SUD. The keywords included were COVID-19, SARS-CoV-2, opioid, opioid use disorder, hospital admission, hospitalization, intensive care, death, mortality, and other related MeSH terms. Additional file 1: Table S1 presents a complete list of the keywords used in the search. Reference screening of the included articles was also performed for possible new included studies.

After eliminating duplicated articles, two authors, AHB and EB, independently screened the titles and abstracts of the articles based on the inclusion criteria. In case of disagreement, the full text of the articles was explored, and any discrepancy was resolved through discussion with BB. A study with the largest dataset was chosen to be included in the systematic review and meta-analysis from articles including the same or nearly the same clinical population.

### Quality assessment

The quality of the included articles was evaluated using the New Castle Ottawa Scale (NOS) designed for observational studies [18]. A NOS score of at least 7 was considered high quality, 5–6 as moderate quality, and less than 5 as low quality. EB and BB independently assessed the quality, and any disagreement was resolved through discussion with a third author (AHB).

### Data extraction

With high consensus levels (kappa coefficient > 0.8), reviewers (EB and BB) independently extracted data from the eligible studies. As independent authors, MF and AHB cross-checked the data extraction and employed a standardized coding protocol to collect data such as country, age, gender, title of study, authors, publication date, study setting, study design, methodology, study population, and rate of outcomes.

### Statistical analysis

The meta‐analysis was performed using Stata software (Stata/MP 16.0, Stata Corp LLC, College Station, Texas, USA). Odds ratio (OR) along with a 95% confidence interval (CI) was used to perform random-effect meta-analysis (DerSimonian-Laird model) for the comparison of each outcome between OUD and non-OUD groups. Cochran’s Q and I^2^ statistics were used based on the random-effects models to determine heterogeneity among the studies. The heterogeneity (I^2^) was classified as mild (25–49%), moderate (50–74%), and high (> 75%) [19]. The funnel plot and statistical examinations of Egger's, Begg's, and "trim and fill" tests were also used to identify potential publication bias [20, 21]. Meta-regression for the quantitative variables, including sample size, NOS score, male percentage, and publication year was conducted in order to identify the source of heterogeneity. The corresponding bubble plots were also designed. Two-tailed p-value < 0.05 was set as the level of statistical significance.

## Results

### Screening the search results and quality assessment

**Fig. 1:**
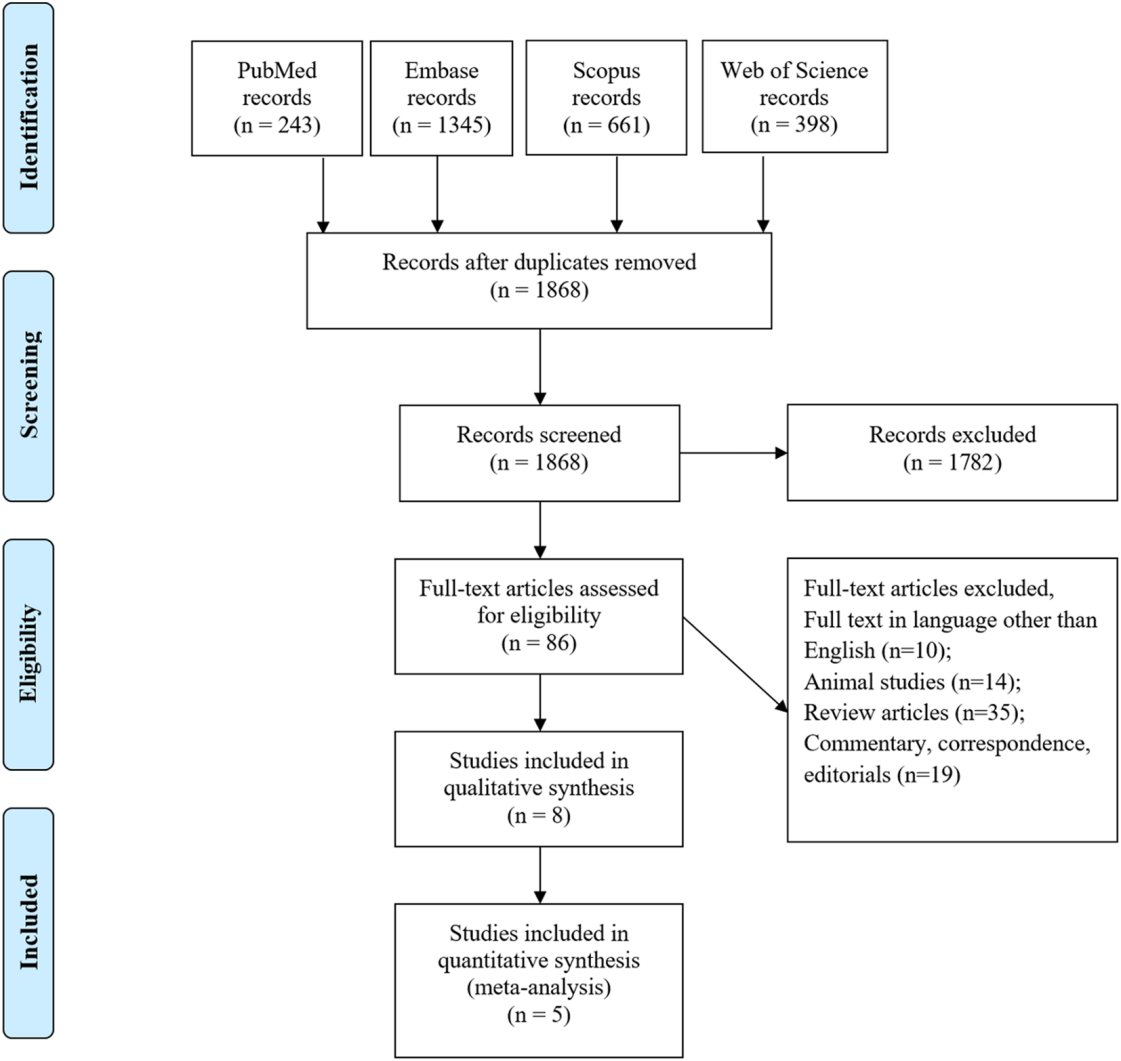
Flow diagram summarizing the selection of eligible studies based on the PRISMA guidelines

According to the selection process of the PRISMA flow diagram in Fig. 1, a total of 2,647 records were identified by searching the databases, which then decreased to 1,868 after removing duplicates. Among these, 1,782 were excluded by title/abstract screening, and 86 were assessed for eligibility based on their full texts. Several articles investigated the complications of total SUD and COVID-19 outcomes did not focus on OUD complications separately. The present study extracted all the data of all studies that investigated total SUD with the inclusion of OUD and analyzed them based on associations between OUD and COVID-19 complications, as reported in the manuscript. Namely, SUD consisted of opioids, drugs, and alcohol (ethanol and methanol) in most of the studies.

Finally, a total of eight articles were included in the systematic review [22–29], of which three did not report OUD-only outcomes separately and reported the outcomes of overall SUD patients [22–24]. Table 1 illustrates the detail and findings of the articles included in the systematic review. The majority of studies were conducted in the United States [22, 23, 25–27], while two were done in Iran [28, 29] and one in Spain [24]. Moreover, the diagnosis of OUD was defined based on the interview [26, 28, 29], or from previously collected registries [23, 27].

**Table 1:** **–** Baseline characteristics of the included studies

Study	Design	Publication year	Study period	Country	Population	Age (years)	Male %	Outcomes	Findings
Allen et al.	Retrospective Cohort	2020	January 1, 2020 – October 26, 2020	United States	Patients tested for COVID-19 between 1 January and 26 October 2020 at four centers in New York City	NR	43	COVID-19 infection, hospitalization, ICU admission, mortality	SUD, alcohol use disorder and OUD were associated with over 2.5 times the odds of ICU admission, and OD with five times the odds [5.00 (3.02–8.30)]. Overdose was associated with mortality [3.03 (1.70–5.43)]
Baillargeon et al.	Retrospective Cohort	2021	February 20, 2020 –June 30, 2020	United States	Adult patients (age > 18) diagnosed as having a COVID-19 infection	54 ± 17.2	52.95	COVID-19 hospitalization, Mortality	Substance use disorder was associated with an increased risk of hospitalization (32.5% versus 17.4%, odds ratio [OR] = 2.29, 95% CI: 2.16–2.44), ventilator use (6.0% versus 3.1%, OR: 2.02, 95% CI: 1.79–2.28) and mortality (4.9% versus 2.8%, OR:1.81, 95% CI:1.58–2.07)
Jamali et al.	Prospective Cohort	2021	February 2020–November 2020	Iran	All COVID-19 patients, including outpatients and inpatients	61.1 ± 6.1	59.3	COVID-19 Infection	The incidence of COVID-19 was 4.47% in the group without OUD and 3.33% in the group with OUD. The relative risk for people with OUD was estimated to be 0.74 (95% CI: 0.28–1.97)
Qeadan et al.	Retrospective Cohort	2021	January 2020–June 2020	United States	Patients included in the sample were identified as having an encounter associated with a diagnosis or recent positive lab result (at the encounter or up to two weeks prior) for COVID-19	51.95 ± 24.46	49.3	COVID-19 hospitalization, mortality	Overall, there was no significant association between having an OUD and odds of death due to COVID-19 (aOR : 1.15, 95% CI : 0.94, 1.41). However, stratifying by age indicated patients younger than 45 with a history of OUD exhibited significantly higher odds of death (aOR: 3.23, 95% CI:1.59, 6.56) than patients without an OUD
Riahi et al.	Cross-Sectional	2021	March 2020–May 2020	Iran	Iranian patients affected by COVID-19	59.35 ± 16.40	57.01	COVID-19 Infection, ICU admission	There was no significant difference between the groups regarding mean days of hospitalization; however, the need for ICU admission was significantly higher in the opium positive group (36.1% vs 11.3% (p:0.005))
Vallecillo et al.	Cross-Sectional	2021	March 12, 202–June 21, 2020	Spain	All individuals (aged ≥ 18 years) with SUD who were admitted for COVID-19 pneumonia	56.1 ± 10.3	85.2	COVID-19 ICU admission	During a median length of stay of 10 days (IQR: 7– 19), severe pneumonia developed in 7(25.9%) patients, acute respiratory distress syndrome in 5 (18.5%) and none died
Velásquez García et al.	Retrospective Cohort	2021	January 26, 2020–January 15, 2021	United States	Individuals who tested positive for SARS-CoV-2 by real-time reverse transcription–polymerase chain reaction (PCR)	NR	51.2	COVID-19 hospitalization	A total of 56,874 COVID-19 cases were investigated and the proportion of individuals with SUD among hospital admissions (13.7%) was higher than in those who did not require hospitalization (4.3%)
Wang et al.	Retrospective Case–Control	2021	Up to July 29, 2020	United States	Population-level electronic health record (EHR) from 360 hospitals in the US	NR	46	COVID-19 infection, hospitalization, mortality	From the total of 73,098,850 individuals 43,160 were OUD. Among 12,030 patients diagnosed with COVID-19, 210 had lifetime OUD (1.75%)

Data are represented as mean ± standard deviation or percentage (%)

*OR Odds ratio, SUD* substance use disorder, *OUD* opioid use disorder, *OD* overdose, *PCR* polymerase chain reaction, *ICU* intensive care unit,* IQR* interquartile range,* NR* not reported

Five studies were included in the meta-analyses [25–29], representing 73,345,758 participants, of whom 49,184 had OUD. The mean age of participants was 57.90 ± 13.4, years and 45.67% of them were male. Out of the five included articles, four reported associations between OUD and COVID-19, two reported data on hospitalization, two reported ICU admission data, and two compared mortalities of COVID-19 patients.

Out of the eight quality-assessed peer-reviewed articles included in the systematic review, seven were rated as high-quality [22, 24–29] and one as moderate-quality [23]. GRADE assessment indicated high certainty for estimating the primary outcome and crude hospitalization and moderate certainty for adjusted hospitalization and ICU admission. Adjusted ICU admission was rated as very low certainty. Failing to properly match the two study groups and the follow-up of shorter than 30 days significantly contributed to reductions in the quality. Additional file 1 Table S2 summarizes the results of assessing the quality of the included articles.

### Outcomes of meta-analysis, sensitivity analysis, publication bias and meta-regression

**Fig. 2:**
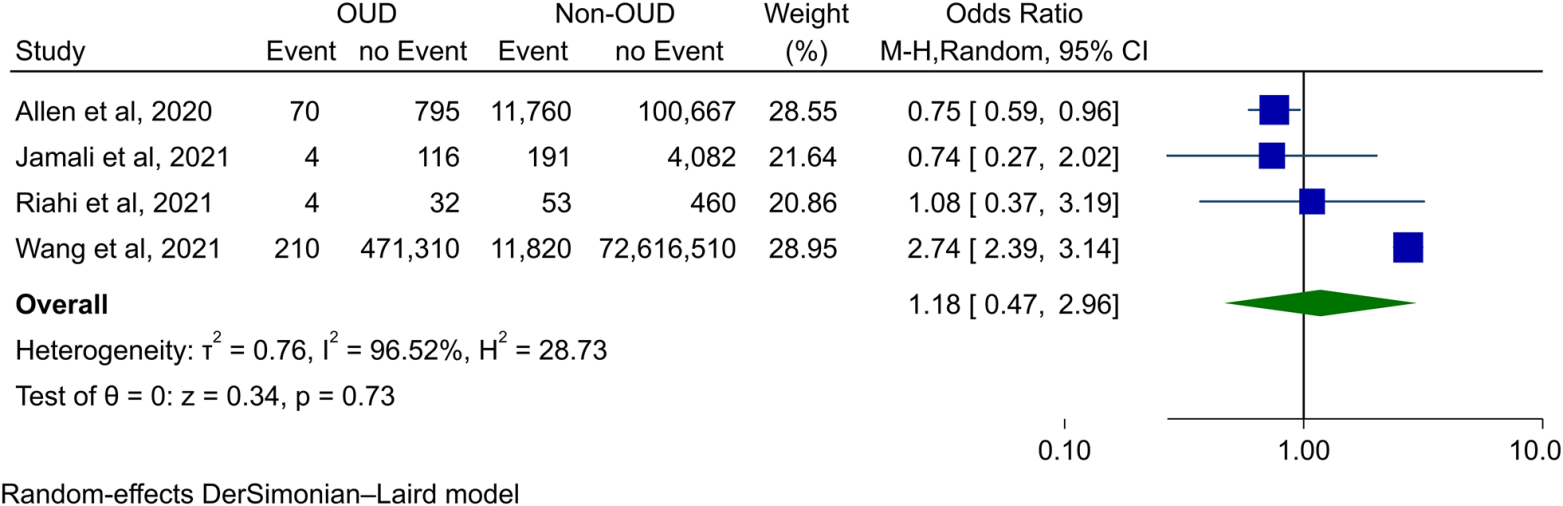
Results of COVID-19 infection meta-analysis

The random-effects analysis of the study outcomes revealed no robust evidence of increased risks of COVID-19 infection in the patients with OUD compared with those without OUD (Fig. 2; OR (95% CI): 1.18 (0.47–2.96); p-value: 0.73). This was associated with high Heterogeneity (I^2^ = 96.5%) for this outcome.

**Fig. 3:**
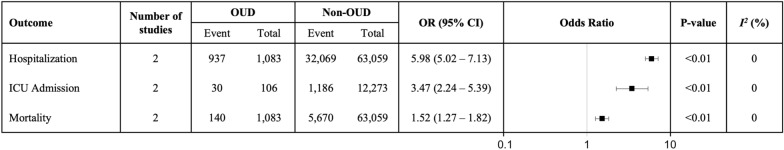
Summary of meta-analyses on COVID-19 outcomes

The results of COVID-19 outcomes are illustrated in Fig. 3. The risk of hospitalization after infection with the disease was significantly higher in the patients with OUD than in those without OUD with no heterogeneity (Additional file 1: Figure S1, OR (95% CI): 5.98 (5.02–7.13); p-value < 0.01). In addition, the pooled OR in the two included articles suggested that OUD increased the risk of ICU admission by more than threefold (Additional file 1: Figure S2, OR (95% CI): 3.47 (2.24–5.39); p-value < 0.01). With regards to mortality, OUD patients had a significantly higher death rate than those without OUD (Additional file 1: Figure S3, OR (95% CI): 1.52 (1.27–1.82); p-value < 0.01) Heterogeneity was low (I^2^ = 0.0%) in hospitalization, ICU admission, and mortality in these models.

Sensitivity analysis showed no significant change in ORs except for omitting Wang et al. (30), which resulted in a statistically significant effect size for COVID-19 infection in favor of OUD patients (OR (95% CI): 0.77 (0.61–0.97); p-value: 0.02).

As there was a high heterogeneity in COVID-19 infection analysis, meta-regression was performed to identify its source. The analyzed variables included sample size, NOS score, publication year, and the male percentage. The sample size was shown to be the only source of heterogeneity, contributing to 100% of it (p-value < 0.001). All other variables did not show any significant contribution to the heterogeneity (Table 2). This represents that the high heterogeneity observed is due to the difference in the sample sizes of the studies. Bubble plots for all these outcomes are shown in Additional file 1: figures S4 and Additional file 1: fig. S5 and Additional file 1: fig. S5 and Additional file 1: fig. S6 and Additional file 1: fig. S7.

**Table 2:** Meta-regression results for the COVID-19 infection outcome

Moderator	Meta-regression				R^2^ Analog (proportion of variance explained) %
	Slope	95% CI		p-value	
Sample size	1.75 $\times$ 10^–8^	1.38 $\times$ 10^–8^	2.11 $\times$ 10^–8^	< 0.001	100
NOS score	0.331	− 0.621	1.283	0.496	0.00
Publication year	0.676	− 0.869	2.221	0.391	0.00
Male Percentage	− 0.024	− 0.143	0.095	0.697	0.00

*NOS* new-castle ottawa scale,* CI* confidence interval

The funnel plot for COVID-19 infection in patients with OUD showed apparent asymmetry, but Egger's and Begg’s tests did not reveal any significant publication bias (p-value : 0.46 and p-value : 00, respectively) (Additional file 1: Figure S8).

## Discussion

Our study assessed the association between OUD and COVID-19 infection in addition to the disease’s complications, including hospitalization, ICU admission, and mortality. Despite the insignificant association between OUD and COVID-19 infection, there was a higher rate of hospitalization in patients with OUD, compared to non-OUD cases. Our meta-analysis reported higher ICU admission rates in these patients; also, mortality was higher in the patients with OUD compared to those without.

A large body of literature suggests the immunosuppressive effects of chronic OUD among people with long-term opioid use (≥ 90 days a year) are exerted through binding to μ-opioid receptors, modulating downstream cellular signal pathways, and impairing the function of virtually immune cells [30–32]. ICU stay has been prolonged, and risks of COVID-19-related hospitalization, respiratory depression, and death have been increased in the globally-growing population of patients with OUD and compromised immune systems.

The included articles showed no significant associations between OUD and COVID-19. Given the controversial effects of OUD on COVID-19, research suggests a higher risk of COVID-19 in patients with substance use disorder or OUD [33–36]. Although not shown in our analysis, there can be several contributing factors for this, including their immunosuppression status caused by opioids, behavioral and economic factors, and living in crowded and closed places. On the other hand, resistance to COVID-19 was reported in tea and tobacco consumers, as well as in patients with OUD [37, 38]. The observation that COVID-19 infection was not statistically different in OUD and non-OUD groups may also stem from the fact that OUD cases are unwilling to appear in the general population or gatherings.

The results of this study showed significant differences between OUD and non-OUD in the aspect of hospitalization, which was reported similarly in several previous studies [15, 36, 39]. Respiratory depression caused by OUD can increase the risk of COVID-19-induced hypoxemia and immunosuppression and subsequently raise vulnerability to COVID-19 outcomes in patients with OUD, which causes a higher hospitalization rate. Since higher male percentage, higher age, and higher frequency of chronic conditions were associated with worse COVID-19 outcomes, such as hospitalization, these may be contributing factors for the results observed in OUD patients [40]. However, the causal relationship between these should be further studied. As there has been higher hospitalization in OUD cases, practical challenges in urgent need for the hospitalization of patients with OUD should be considered by healthcare systems, including comorbidities such as older age, cancer, psychiatric disorders (and the effect of their medications), and chronic pain [41–43], in addition to a higher risk of end-organ damage [44, 45]. Higher ICU admission and mortality rates observed in patients with OUD can also have clinical implications, including focusing on monitoring during the hospitalization course. In addition, healthcare providers anticipate long-term treatments and invasive procedures such as intubation in these patients. The adverse effects of opioids on the respiratory system and the associated increased histamine release caused by OUD also cause extubating challenges in these patients [30–32, 46, 47]. Moreover, medications used preliminary for COVID-19, such as hydroxychloroquine and azithromycin, although the efficacy could not be later established in studies [48, 49], significantly interact with certain opioids, including methadone [50, 51]. Clinicians should be, therefore, aware of the co-prescription of drugs and medicines used for opioid withdrawal in inpatients.

This meta-analysis suggested associations between OUD and ICU admission after adjusting for other risk factors in the included articles, such as age, sex, race, COVID-19 medications, and some underlying conditions like cancer, hypertension, diabetes, immunosuppression, and asthma, which can be attributed to different comorbidities and complications in the individual patients. Given the controversial relationships between ICU admission and OUD [52–54], there may be the chance of low-quality study or wide range for OR which can affect the overall results and should be considered in future studies.

In line with the literature, the present research showed increased risks of COVID-19-associated mortality in patients with OUD, which can stem from biological processes such as immunosuppression and elevated cytokine concentration [22, 27, 55]. Saeedi et al. reported significant associations between OUD and mortality in patients with COVID-19 (OR (95% CI): 3.59, (0.9–14.5)) [34]. Furthermore, OUD was reported as a contributing factor to increasing several infectious diseases such as HIV, endocarditis, hepatitis, and some other viral infections [56].

The higher hospitalization, ICU admission, and mortality in OUD patients should be taken into consideration in the vaccination programs. These programs have been implemented globally, and the efficacy of vaccines in terms of all investigated factors (infection, hospitalization, ICU admission, and mortality) has been shown in several studies [57–59]. Given the higher COVID-19 complications in OUD cases, preventive measures such as vaccination should be especially considered in them in order to reduce the COVID-19 burden in this highly susceptible population. This is of more importance as there has been a report that complacency and convenience were the main barriers to COVID-19 vaccination in people with OUD [60].

## Study strengths and limitations

This study provided evidence for evaluating associations between OUD and COVID-19 outcomes. Several studies evaluated the effects of SUDs on COVID-19 outcomes; nevertheless, OUD has been less addressed individually in the literature. The low number of included articles, therefore, constituted a limitation of the present study. Given the several low-quality articles or those with a small sample, the evidence obtained for COVID-19 outcomes was of low certainty. Moreover, the observational nature of studies and the potential risk of confounding biases, in addition to the low number of studies for some of the outcomes, could have an impact on the overall pooled result. Further evidence is therefore required for determining the accuracy and generalizability of the present findings, especially ICU admission, based on relevant factors and comorbidities. Importantly, this study focuses on the early phase of the COVID-19 pandemic and, therefore, may have been affected during the next phases and introduction of vaccines. In many countries, it can be applicable and provide valuable information regarding OUD and COVID-19. The diverse definitions provided for OUD also decreased the number of included articles; nevertheless, efforts were made to uniformly define OUD in the present study, especially in the context of pandemics.

## Conclusion

Our meta-analysis revealed that OUD was not associated with COVID-19 infection; however, patients with OUD were more likely to be hospitalized and receive treatment in ICU. Moreover, according to the meta-analysis, mortality was reported to be higher in the patients with OUD compared to the other patients. Therefore, as this population is at higher risk of suffering and complications from the COVID-19 pandemic, policymakers and healthcare systems should pay special attention to them.

## Supplementary Information

**Additional file 1: Table S1.** Keywords used for search in databases. **Table S2.** Newcastle Ottawa Scale for Quality Assessment. **Figure S1.** Results of COVID-19 hospitalization outcome meta-analysis. **Figure S2.** Results of COVID-19 ICU admission outcome meta-analysis. **Figure S3.** Results of COVID-19 mortality outcome meta-analysis. Figur**e S4.** Bubble plot of meta-regression of sample size. **Figure S5.** Bubble plot of meta-regression of NOS score. **Figure S6.** Bubble plot of meta-regression of publication year. **Figure S7.** Bubble plot of meta-regression of male percentage. **Figure S8.** Funnel plot for COVID-19 infection in patients with OUD.

## Data Availability

Not applicable.

## Abbreviations

COVID-19: Coronavirus disease-2019
OUD: Opioid use disorder
SARS-CoV-2: Severe acute respiratory syndrome coronavirus 2
SUD: Substance use disorder
ICU: Intensive care unit
NOS: Newcastle–Ottawa Scale
PCR: Polymerase chain reaction
PRISMA: Preferred reporting items for systematic reviews and meta-analyses

## References

[1] WHO. Listings of WHO’s response to COVID-19 2020. https://www.who.int/news/item/29-06-2020-covidtimeline.

[2] WHO. WHO coronavirus (COVID-19) dashboard. https://covid19.who.int

[3] Behnoush AH, Ahmadi N, Mozafar M, Mirghaderi SP, Azad AJ, Houjaghan AK, et al. Anxiety, depression, and their contributing factors among nurses infected with COVID-19 in Iran: a cross-sectional study. Iranian Red Crescent Med J. 2022;24(2).

[4] Cummings MJ, Baldwin MR, Abrams D, Jacobson SD, Meyer BJ, Balough EM (2020). Epidemiology, clinical course, and outcomes of critically ill adults with COVID-19 in New York city: a prospective cohort study. Lancet.

[5] Bandyopadhyay S, Baticulon RE, Kadhum M, Alser M, Ojuka DK, Badereddin Y (2020). Infection and mortality of healthcare workers worldwide from COVID-19: a systematic review. BMJ Global Health.

[6] Shali M, Behnoush AH, Shabani EA, Khazaeipour Z (2022). Individual and working experiences of healthcare workers infected with COVID-19 A qualitative study. Japan J Nursing Sci.

[7] Global, regional, and national incidence, prevalence, and years lived with disability for 354 diseases and injuries for 195 countries and territories, 1990–2017: a systematic analysis for the global burden of disease study 2017. Lancet 2018 392(10159):1789–858. 10.1016/S0140-6736(18)32279-7 PMC6227754 30496104

[8] Schimmel J, Manini AF (2020). Opioid use disorder and COVID-19: biological plausibility for worsened outcomes. Subst Use Misuse.

[9] Mansuri Z, Shah B, Trivedi C, Beg U, Patel H, Jolly T (2020). Opioid use disorder treatment and potential interactions with novel COVID-19 medications: a clinical perspective. Primary Care Companion CNS Dis.

[10] Alexander GC, Stoller KB, Haffajee RL, Saloner B (2020). An epidemic in the midst of a pandemic: opioid use disorder and COVID-19. Ann Intern Med.

[11] Becker WC, Fiellin DA (2020). When epidemics collide: coronavirus disease 2019 (COVID-19) and the opioid crisis. Ann Intern Med.

[12] Qeadan F, Mensah NA, Tingey B, Bern R, Rees T, Madden EF (2021). The association between opioids, environmental, demographic, and socioeconomic indicators and COVID-19 mortality rates in the United States: an ecological study at the county level. Archiv Public Health.

[13] Fond G, Pauly V, Leone M, Llorca PM, Orleans V, Loundou A (2021). Disparities in intensive care unit admission and mortality among patients with Schizophrenia and COVID-19: a national cohort study. Schizophr Bull.

[14] Jeon HL, Kwon JS, Park SH, Shin JY (2021). Association of mental disorders with SARS-CoV-2 infection and severe health outcomes: nationwide cohort study. Br J psychiatry J Mental Sci.

[15] Wei Y, Shah R (2020). Substance use disorder in the COVID-19 pandemic a systematic review of vulnerabilities and complications. Pharmaceuticals.

[16] Kumar N, Janmohamed K, Nyhan K, Martins SS, Cerda M, Hasin D (2021). Substance use and substance use disorder, in relation to COVID-19: protocol for a scoping review. Syst Rev.

[17] Moher D, Liberati A, Tetzlaff J, Altman DG (2009). Preferred reporting items for systematic reviews and meta-analyses: the PRISMA statement. BMJ.

[18] GA Wells BS, D O'Connell, J Peterson, V Welch, M Losos, P Tugwell. The Newcastle-Ottawa Scale (NOS) for assessing the quality of nonrandomised studies in meta-analyses. 2000

[19] Higgins JP, Thompson SG, Deeks JJ, Altman DG (2003). Measuring inconsistency in meta-analyses. BMJ.

[20] Begg CB, Mazumdar M (1994). Operating characteristics of a rank correlation test for publication bias. Biometrics.

[21] Egger M, Davey Smith G, Schneider M, Minder C (1997). Bias in meta-analysis detected by a simple, graphical test. BMJ.

[22] Baillargeon J, Polychronopoulou E, Kuo YF, Raji MA (2021). The Impact of substance use disorder on COVID-19 outcomes. Psychiatr Serv.

[23] Velásquez García HA, Wilton J, Smolina K, Chong M, Rasali D, Otterstatter M (2021). Mental health and substance use associated with hospitalization among people with COVID-19: a population-based cohort study. Viruses.

[24] Vallecillo G, Perelló R, Güerri R, Fonseca F, Torrens M (2021). Clinical impact of COVID-19 on people with substance use disorders. J Public Health.

[25] Allen B, El Shahawy O, Rogers ES, Hochman S, Khan MR, Krawczyk N (2021). Association of substance use disorders and drug overdose with adverse COVID-19 outcomes in New York city: january-october 2020. J Public Health (Oxf).

[26] Qeadan F, Tingey B, Bern R, Porucznik CA, English K, Saeed AI (2021). Opioid use disorder and health service utilization among COVID-19 patients in the US: a nationwide cohort from the cerner real-world data. EClinicalMedicine.

[27] Wang QQ, Kaelber DC, Xu R, Volkow ND (2021). COVID-19 risk and outcomes in patients with substance use disorders: analyses from electronic health records in the United States. Mol Psychiatry.

[28] Jamali Z, Emamian MH, Hashemi H, Fotouh A (2021). The association of opioid use disorder and COVID-19 in shahroud Iran. medrxiv..

[29] Riahi T, Sadeghzadeh-Bazargan A, Shokri S, Ahmadvand D, Hassanlouei B, Baghestani A (2021). The effect of opium on severity of COVID-19 infection: an original study from Iran. Med J Islam Repub Iran.

[30] Joseph DPR, Krovvidi H (2013). Non-respiratory functions of the lung. Contin Educ Anaesth Crit Care Pain.

[31] Eisenstein TK (2019). The role of opioid receptors in immune system function. Front Immunol.

[32] Plein LM, Rittner HL (2018). Opioids and the immune system—friend or foe. Br J Pharmacol.

[33] Farhoudian A, Baldacchino A, Clark N, Gerra G, Ekhtiari H, Dom G (2020). COVID-19 and substance use disorders: recommendations to a comprehensive healthcare response an international society of addiction medicine practice and policy interest group position paper. Basic Clin Neurosci.

[34] Saeedi M, Omrani-Nava V, Maleki I, Hedayatizadeh-Omran A, Ahmadi A, Moosazadeh M (2020). Opium addiction and COVID-19: truth or false beliefs. Iran J Psychiatry Behav Sci.

[35] Volkow ND (2020). Collision of the COVID-19 and addiction epidemics. Ann Intern Med.

[36] Melamed OC, Hauck TS, Buckley L, Selby P, Mulsant BH (2020). COVID-19 and persons with substance use disorders: Inequities and mitigation strategies. Subst abuse.

[37] de Bernardis E, Busà L (2020). A putative role for the tobacco mosaic virus in smokers' resistance to COVID-19. Med Hypotheses.

[38] Mhatre S, Srivastava T, Naik S, Patravale V (2021). Antiviral activity of green tea and black tea polyphenols in prophylaxis and treatment of COVID-19: a review. Phytomed Int J Phytother Phytopharmacol.

[39] Enns A, Pinto A, Venugopal J, Grywacheski V, Gheorghe M, Kakkar T (2020). Substance use and related harms in the context of COVID-19: a conceptual model. Health Promot Chron Dis Prevention Canada Res Policy Pract.

[40] Zheng Z, Peng F, Xu B, Zhao J, Liu H, Peng J (2020). Risk factors of critical & mortal COVID-19 cases: a systematic literature review and meta-analysis. J Infect.

[41] Namba RS, Singh A, Paxton EW, Inacio MCS (2018). Patient factors associated with prolonged postoperative opioid use after total knee arthroplasty. J Arthroplasty.

[42] Kobus AM, Smith DH, Morasco BJ, Johnson ES, Yang X, Petrik AF (2012). Correlates of higher-dose opioid medication use for low back pain in primary care. J Pain.

[43] Buckeridge D, Huang A, Hanley J, Kelome A, Reidel K, Verma A (2010). Risk of injury associated with opioid use in older adults. J Am Geriatr Soc.

[44] Ataei M, Shirazi FM, Lamarine RJ, Nakhaee S, Mehrpour O (2020). A double-edged sword of using opioids and COVID-19: a toxicological view. Subst Abuse Treat Prev Policy.

[45] Porubsky S, Kuppe C, Maier T, Birk HW, Wörnle M, Moeller MJ (2014). Renal lipidosis in patients enrolled in a methadone substitution program. Arch Pathol Lab Med.

[46] Yamanaka T, Sadikot RT (2013). Opioid effect on lungs. Respirology.

[47] Roy S, Ninkovic J, Banerjee S, Charboneau RG, Das S, Dutta R (2011). Opioid drug abuse and modulation of immune function: consequences in the susceptibility to opportunistic infections. J Neuroimmune Pharmacol Off J Soc NeuroImmune Pharmacol.

[48] García-Albéniz X, del Amo J, Polo R, Morales-Asencio JM, Hernán MA (2022). Systematic review and meta-analysis of randomized trials of hydroxychloroquine for the prevention of COVID-19. Eur J Epidemiol.

[49] Ayerbe L, Risco-Risco C, Forgnone I, Pérez-Piñar M, Ayis S (2022). Azithromycin in patients with COVID-19: a systematic review and meta-analysis. J Antimicrob Chemother.

[50] Chang KC, Huang CL, Liang HY, Chang SS, Wang YC, Liang WM (2012). Gender-specific differences in susceptibility to low-dose methadone-associated QTc prolongation in patients with heroin dependence. J Cardiovasc Electrophysiol.

[51] Fonseca F, Marti-Almor J, Pastor A, Cladellas M, Farré M, de la Torre R (2009). Prevalence of long QTc interval in methadone maintenance patients. Drug Alcohol Depend.

[52] Kim L, Garg S, O'Halloran A, Whitaker M, Pham H, Anderson EJ (2021). Risk factors for intensive care unit admission and in-hospital mortality among hospitalized adults identified through the US coronavirus Disease 2019 (COVID-19)-associated hospitalization surveillance network (COVID-NET). Clin Infect Dis Off publ Infect Dis Soc Am.

[53] Petrilli CM, Jones SA, Yang J, Rajagopalan H, O'Donnell L, Chernyak Y (2020). Factors associated with hospital admission and critical illness among 5279 people with coronavirus disease 2019 in New York city: prospective cohort study. BMJ.

[54] Reilev M, Kristensen KB, Pottegård A, Lund LC, Hallas J, Ernst MT (2020). Characteristics and predictors of hospitalization and death in the first 11 122 cases with a positive RT-PCR test for SARS-CoV-2 in Denmark: a nationwide cohort. Int J Epidemiol.

[55] Balaram K, Marwaha R, Kaelber DC (2021). The effects of substance use on severe acute respiratory syndrome coronavirus infection risks and outcomes. Curr Opin Psychiatry.

[56] Schwetz TA, Calder T, Rosenthal E, Kattakuzhy S, Fauci AS (2019). Opioids and infectious diseases: a converging public health crisis. J Infect Dis.

[57] Thompson MG, Stenehjem E, Grannis S, Ball SW, Naleway AL, Ong TC (2021). Effectiveness of covid-19 vaccines in ambulatory and inpatient care settings. N Engl J Med.

[58] Tenforde MW, Self WH, Adams K, Gaglani M, Ginde AA, McNeal T (2021). Association between mRNA vaccination and COVID-19 hospitalization and disease severity. JAMA.

[59] Rzymski P, Kasianchuk N, Sikora D, Poniedziałek B (2022). COVID-19 vaccinations and rates of infections, hospitalizations, ICU admissions, and deaths in Europe during SARS-CoV-2 Omicron wave in the first quarter of 2022. J Med Virol.

[60] Vallecillo G, Durán X, Canosa I, Roquer A, Martinez MC, Perelló R (2022). COVID-19 vaccination coverage and vaccine hesitancy among people with opioid use disorder in Barcelona. Spain Drug Alcohol Rev.

