# Supplementary material for: Risk of COVID-19 infection and the associated hospitalization, ICU admission and mortality in opioid use disorder: a systematic review and meta-analysis
Source: Addict Sci Clin Pract. 2022 Nov 30;17:68. doi: 10.1186/s13722-022-00349-8 (PMC9709364; doi:10.1186/s13722-022-00349-8)
Supplement: Supplementary file 1 — Additional file 1: Table S1. Keywords used for search in databases. Table S2. Newcastle Ottawa Scale for Quality Assessment. Figure S1. Results of COVID-19 hospitalization outcome meta-analysis. Figure S2. Results of COVID-19 ICU admission outcome meta-analysis. Figure S3. Results of COVID-19 mortality outcome meta-analysis. Figure S4. Bubble plot of meta-regression of sample size. Figure S5. Bubble plot of meta-regression of NOS score. Figure S6. Bubble plot of meta-regression of publication year. Figure S7. Bubble plot of meta-regression of male percentage. Figure S8. Funnel plot for COVID-19 infection in patients with OUD. [file 13722_2022_349_MOESM1_ESM.docx]

**Additional file 1: Table S1 – Keywords used for search in databases**

| **PubMed** |
| --- |
| ("narcotics"[Mesh] OR "Narcotic-Related Disorders" [Mesh] OR "narcotics"[tiab] OR "Opiate Overdose" [Mesh] OR "opiate"[tiab] OR "opioid"[tiab] OR "papaverine"[tiab] OR "Papaverine"[Mesh] OR "narcotic-related disorders" [tiab] OR "Analgesics, Opioid"[Mesh]) AND ("COVID-19"[Mesh] OR "coronavirus "[tiab] OR "coronavirus "[Mesh] OR " severe acute respiratory syndrome coronavirus 2"[tiab] OR "SARS-CoV-2 " [tiab] OR "SARS-CoV-2 "[Mesh] OR "novel coronavirus "[tiab] OR " nCoV "[tiab] OR "2019-nCoV "[tiab] OR " COVID-19"[tiab]) |
| **Scopus** |
| ( ( TITLE-ABS-KEY ( coronavirus ) OR TITLE-ABS-KEY ( "severe acute respiratory syndrome coronavirus 2" ) OR TITLE-ABS-KEY ( "SARS-CoV-2" ) OR TITLE-ABS-KEY ( "novel coronavirus" ) OR TITLE-ABS-KEY ( ncov ) OR TITLE-ABS-KEY ( "2019-nCoV" ) OR TITLE-ABS-KEY ( "COVID-19" ) OR TITLE-ABS-KEY ( covid ) OR TITLE-ABS-KEY ( covid 19 ) ) AND ( TITLE-ABS-KEY ( opioid ) OR TITLE-ABS-KEY ( "opioid overdose" ) OR TITLE-ABS-KEY ( "opiate" ) OR TITLE-ABS-KEY ( "opiate overdose" ) OR TITLE-ABS-KEY ( "papaverine" ) ) OR TITLE-ABS-KEY ( " narcotics" ) ) |
| **Web of science** |
| TS= (coronavirus OR "Covid 19" OR COVID-19 OR "severe acute respiratory syndrome coronavirus 2" OR SARS-CoV-2 OR "novel coronavirus" OR nCoV OR 2019-nCoV) AND TS= (Opioid OR Opiate OR Papaverine OR narcotics) |
| **Embase** |
| ('coronavirus disease 2019'/exp OR 'coronavirus disease 2019':ti,ab,kw OR 'severe acute respiratory syndrome coronavirus 2'/exp OR 'covid-19':ti,ab,kw OR 'coronavirus':ti,ab,kw OR 'severe acute respiratory syndrome coronavirus 2':ti,ab,kw OR 'sars-cov-2':ti,ab,kw OR 'novel coronavirus':ti,ab,kw OR 'ncov' OR '2019-ncov':ti,ab,kw) AND ('opiate'/exp OR 'opiate':ti,ab,kw OR 'opioid':ti,ab,kw OR 'narcotic agent'/exp OR 'narcotic agent':ti,ab,kw OR 'papaverine'/exp OR 'papaverine':ti,ab,kw) |

**Additional file 1: Table S2 – Newcastle Ottawa Scale for Quality Assessment**

| **Study** | **Selection** | | | | **Comparability** | **Exposure/Outcome** | | | **Overall**  **Score** |
| --- | --- | --- | --- | --- | --- | --- | --- | --- | --- |
|  | Representation | Control | Exposure | Outcome |  | Outcome | Follow-up | Lost to Follow-up |  |
| Allen et al. | * | * | * | * | ** | * | * | * | 9 |
| Baillargeon et al. | * | * | * | * | - | * | * | - | 7 |
| Jamali et al. | * | * | * | * | - | * | * | * | 7 |
| Qeaden et al. | * | * | * | * | * | * | * | * | 8 |
| Riahi et al. | * | * | * | * | * | * | * | * | 8 |
| Vallecillo et al. | * | * | * | * | * | * | * | - | 7 |
| Velásquez García et al. | - | - | * | * | * | - | * | * | 5 |
| Wang et al. | * | * | * | * | ** | * | * | * | 9 |


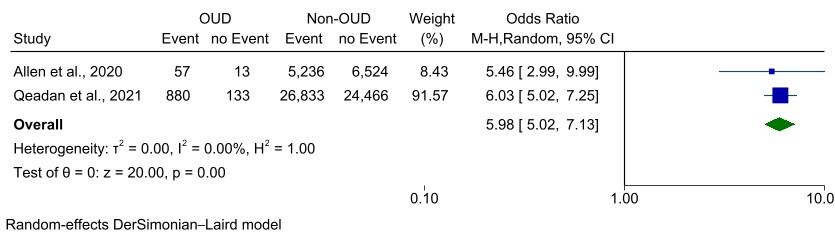


**Additional file 1: Figure S1 – Results of COVID-19 hospitalization outcome meta-analysis**

**
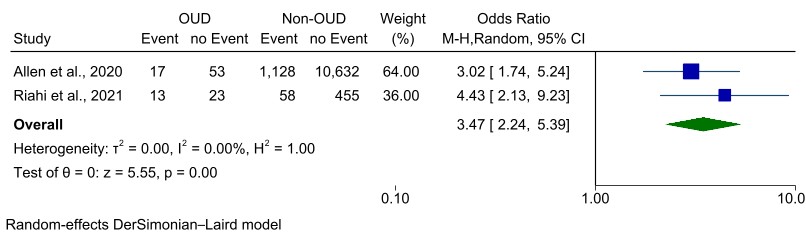
**

**Additional file 1: Figure S2 – Results of COVID-19 ICU admission outcome meta-analysis**

**
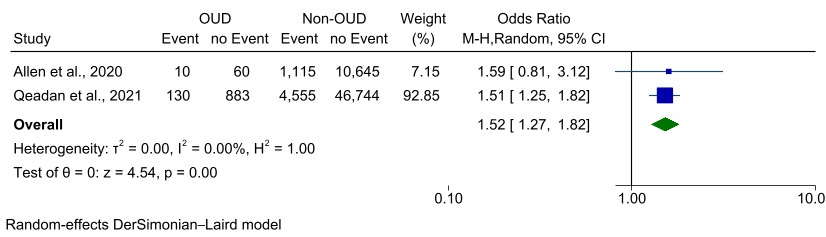
**

**Additional file 1: Figure S3 – Results of COVID-19 mortality outcome meta-analysis**

**
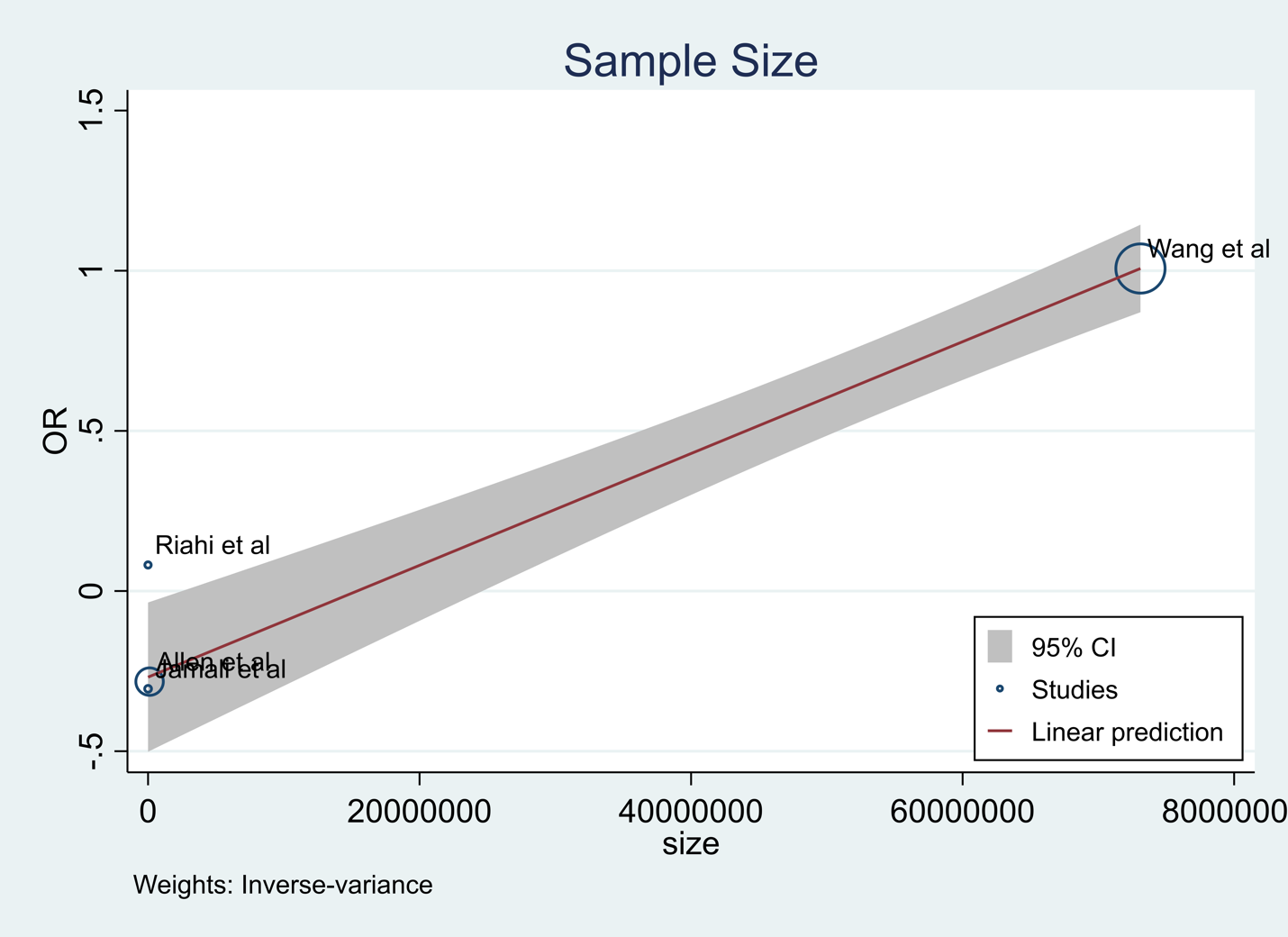
**

**Additional file 1: Figure S4 – Bubble plot of meta-regression of sample size**

**
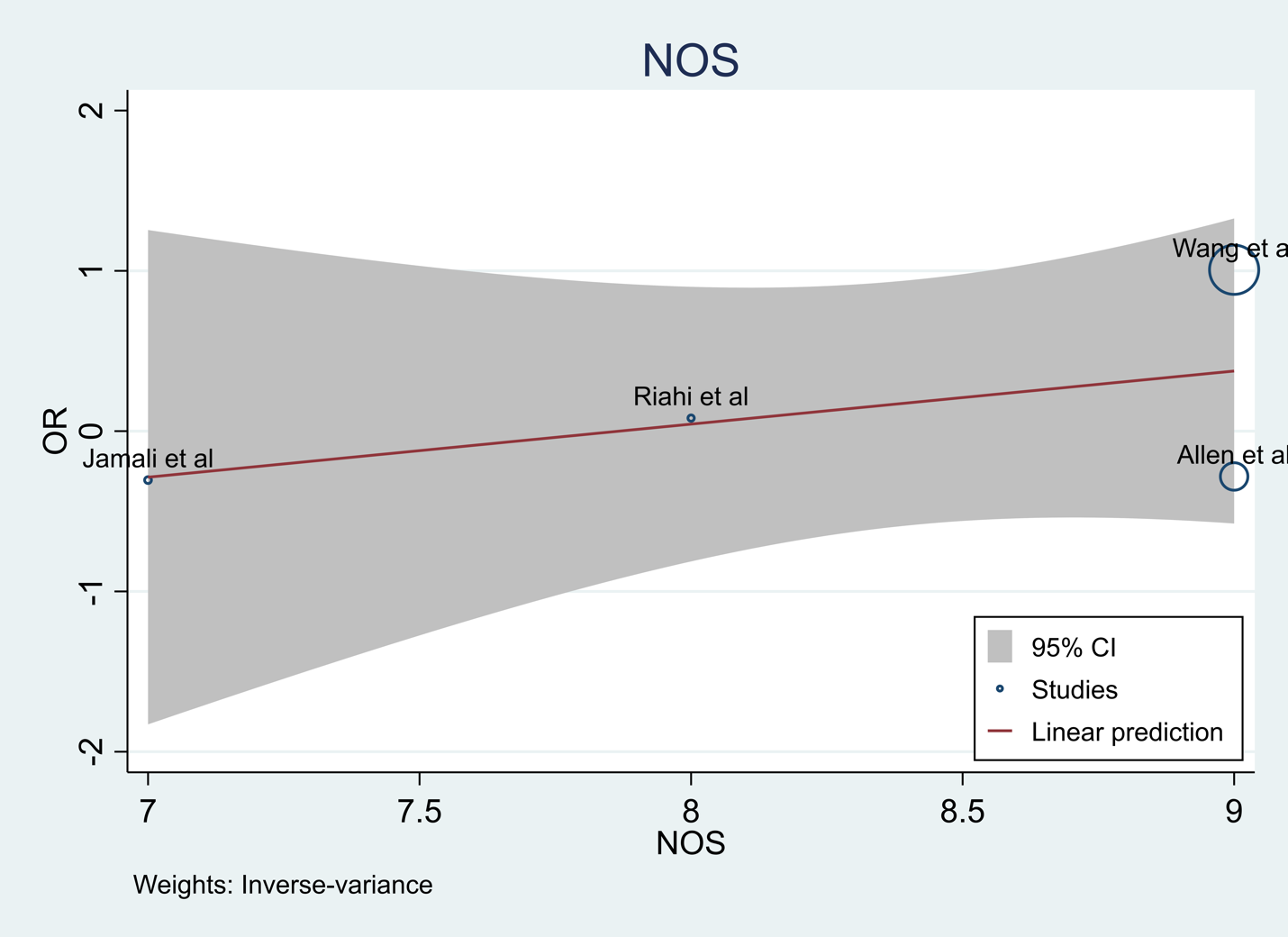
**

**Additional file 1: Figure S5 – Bubble plot of meta-regression of NOS score**

**
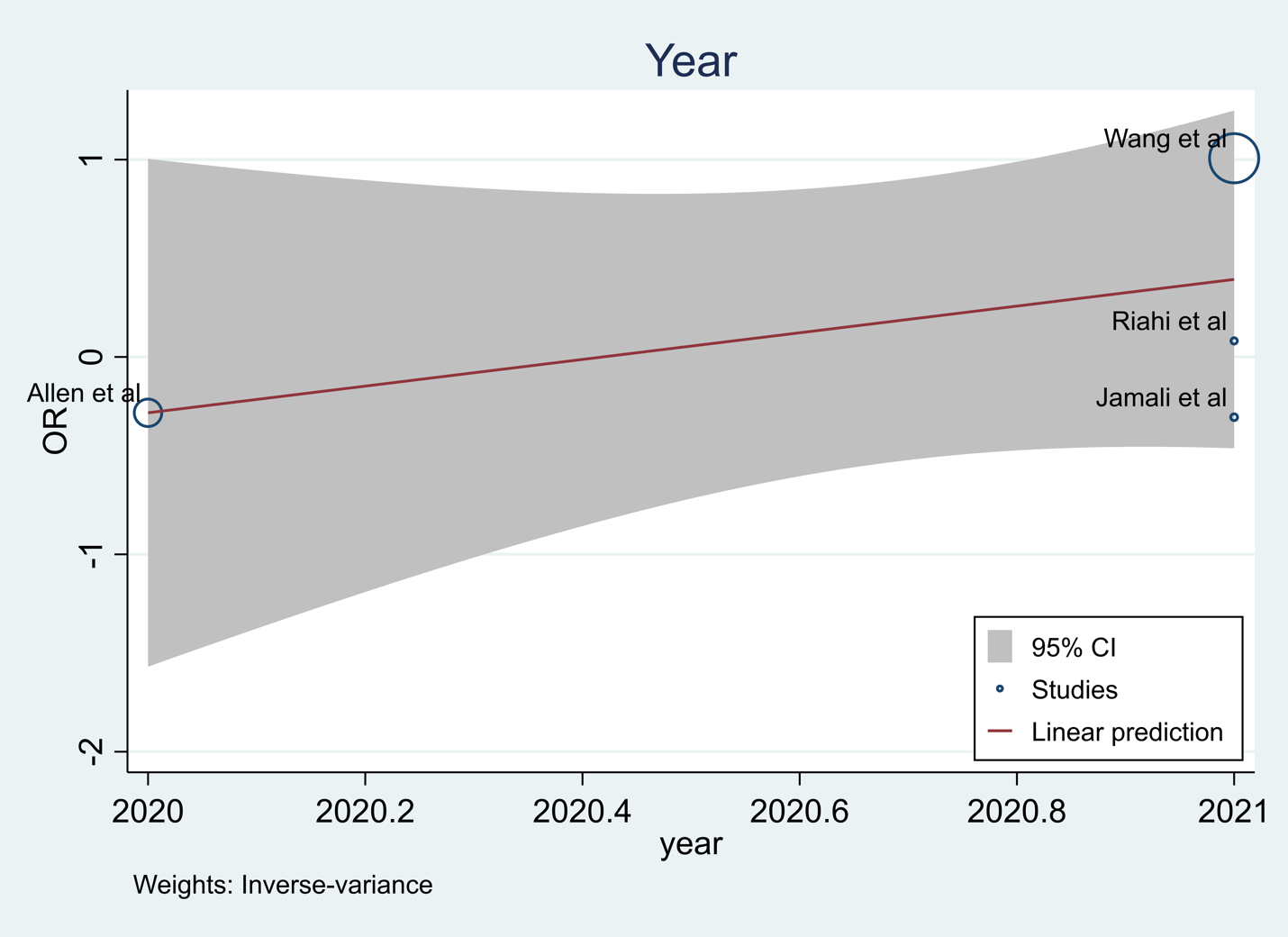
**

**Additional file 1: Figure S6 – Bubble plot of meta-regression of publication year**

**
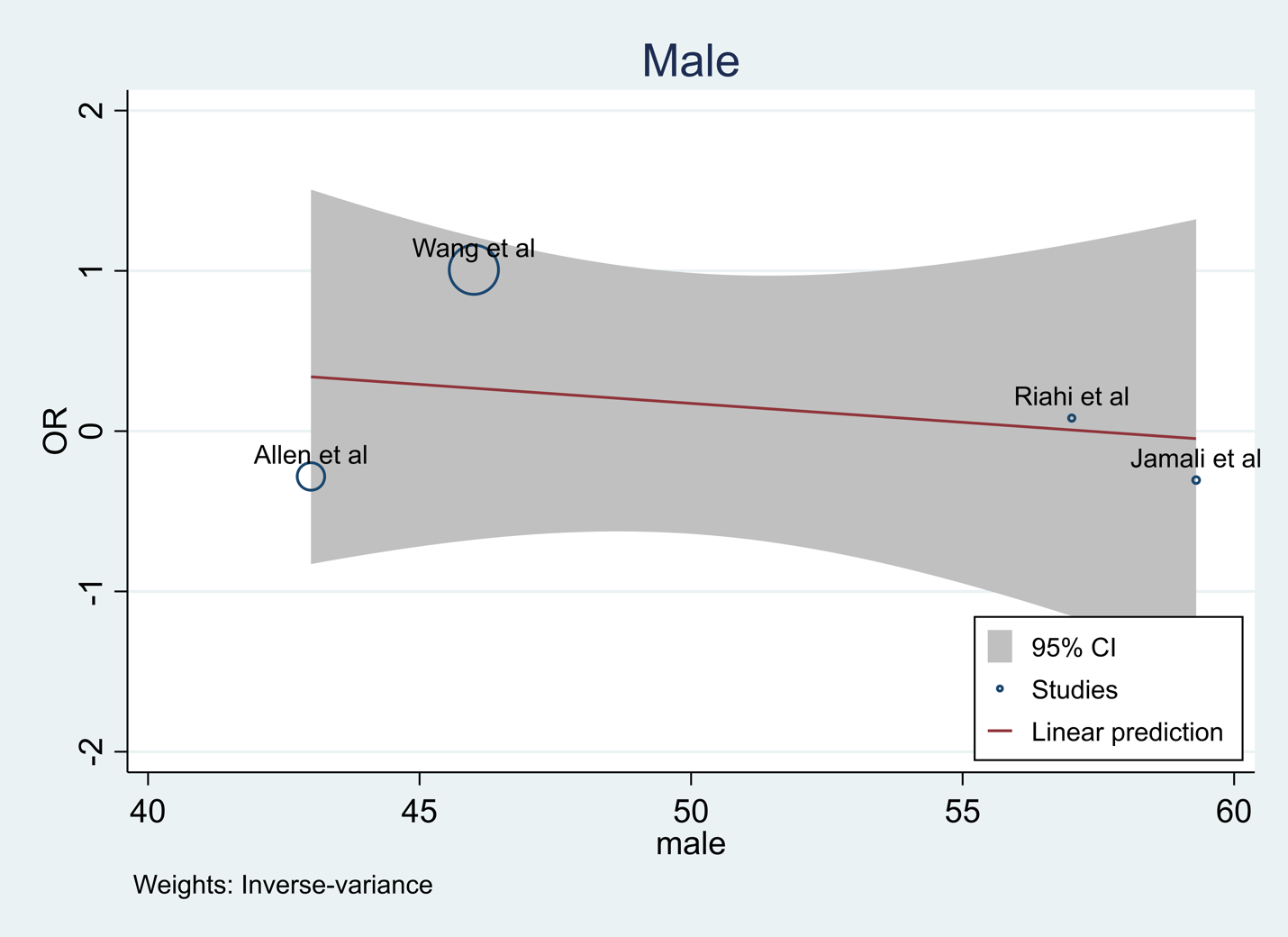
**

**Additional file 1: Figure S7 – Bubble plot of meta-regression of male percentage**

**
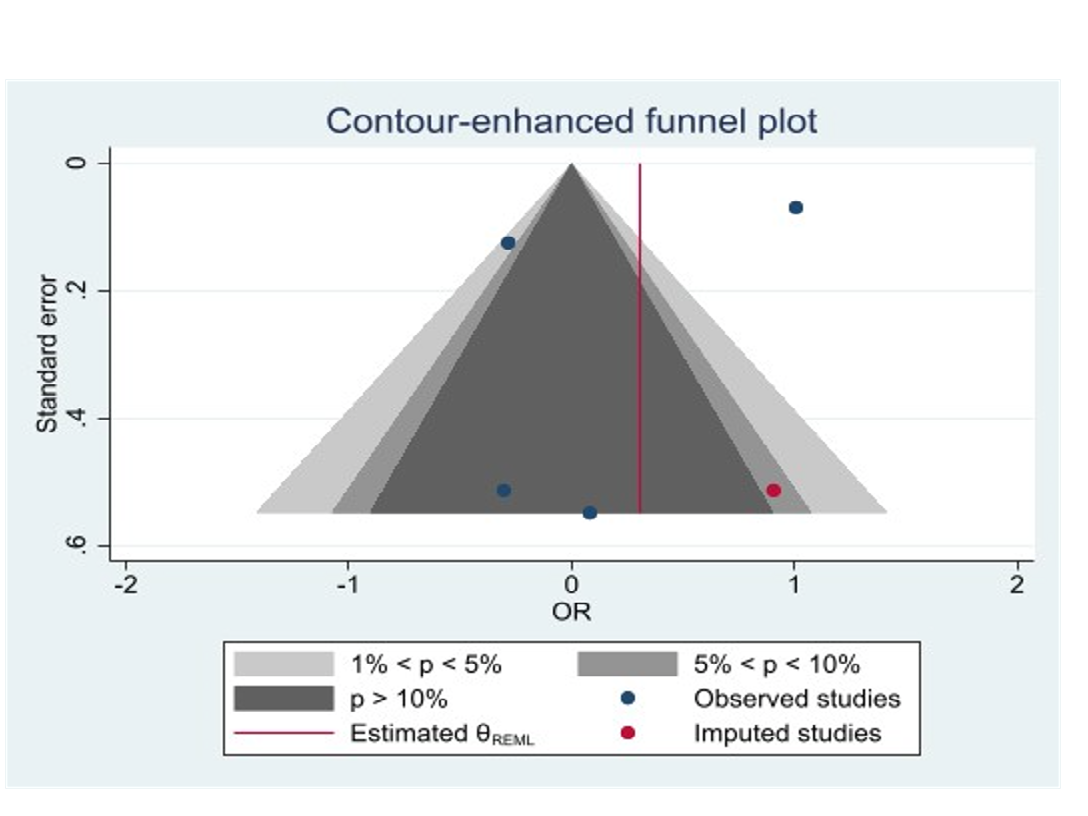
**

**Additional file 1: Figure S8. Funnel plots for COVID-19 infection in patients with OUD**
